# Challenges and Social Implications of Informal Caregiving for People with Alzheimer’s: A Qualitative Study

**DOI:** 10.3390/healthcare13243271

**Published:** 2025-12-12

**Authors:** Andrea Alcaraz-Córdoba, María López-Cano, Olivia Ibáñez-Masero, María Isabel Ventura-Miranda, María Dolores Ruiz-Fernández, Angela María Ortega-Galán

**Affiliations:** 1 Department of Nursing, Physiotherapy and Medicine, University of Almería, 04120 Almería, Spain; 2 Torrecardenas University Hospital, Andalusian Health Service, 04009 Almería, Spain; 3 Almería Health District, Andalusian Health Service, 04009 Almería, Spain; 4 Department of Nursing, University of Huelva, 21071 Huelva, Spain; 5 Facultad de Ciencias de la Salud, Universidad Autónoma de Chile, Providencia 4780000, Chile

**Keywords:** aged adult, Alzheimer disease, compassion, informal caregivers, older adult, social support

## Abstract

**Aim**: The aim of this study was to explore the experiences and challenges faced by informal caregivers of people with Alzheimer’s, including the social and emotional aspects of their caregiving role. **Methods**: A descriptive qualitative study was conducted using one focus group discussion and eleven semi-structured interviews with informal caregivers of individuals diagnosed with Alzheimer’s disease. The data collected were analyzed through thematic analysis using ATLAS.ti qualitative software version 23. **Results**: The results reveal two themes: (1) “Life centred on compassionate care for the other person”, which reflects the role performed from a perspective of emotional and compassionate commitment to those in need of care, and (2) “Abandonment by caregivers”, which expresses the emotional cost associated with caregiving. **Conclusions**: Informal caregivers of people with Alzheimer’s disease undertake their roles guided by compassion, which involves substantial personal sacrifice. This commitment often leads to self-abandonment, impacting their emotional and physical health, social relationships, and personal aspirations. It is therefore crucial to implement psychosocial interventions grounded in compassion and to strengthen both formal and informal social support systems for caregivers.

## 1. Introduction

Alzheimer’s disease is a chronic, progressive, neurodegenerative disease [1] characterized by a wide range of neuropsychiatric symptoms such as delusions, hallucinations, insomnia, functional decline, and the development of behavioural disorders [2,3]. Currently, approximately 50 million people are living with dementia worldwide, and this number is expected to increase to 82 million by 2030 and 152 million by 2050 [4]. It is an emerging public health problem associated with the ageing of the population [5,6] and is considered one of the most costly, lethal, and socially burdensome diseases of the 21st century [7]. Alzheimer’s patient care is typically provided by family members or individuals with a close relationship to the patient, playing the role of primary caregiver [8,9] for daily activities without receiving remuneration [10]. This unpaid contribution represents an important component of social value, understood as the positive changes generated in people’s lives when tangible and intangible resources are mobilized within the community to produce meaningful social outcomes [11,12]. The profile of these caregivers is usually that of a middle-aged woman, daughter or wife of the patient [13] who quits her job due to the responsibilities of caregiving [14,15], which is a great challenge for the family members and friends of those with Alzheimer’s [16,17].

The burden of the caregiver has been defined as “a state of emotional exhaustion, stress, and fatigue associated with the experience of caregiving” [18]. While the objective caregiver burden refers to the amount of time spent on caregiving, the subjective burden indicates how informal caregivers experience the performance of their caregiving tasks [19]. A significant impact on the physical condition, mental health [20], well-being, and social relationships of informal caregivers of people with Alzheimer’s has been described by He et al. [4] and Travis et al. [21]. Other associated symptoms include social dysfunction, anxiety and insomnia [22,23], major depression and an increase in chronic diseases [10]. The development of compassion and empathy has shown positive outcomes in terms of the emotional impact and coping mechanisms of informal caregivers [24,25]. Although the terms compassion and empathy have been used interchangeably in the scientific literature, they are independent constructs [26]. Compassion is defined as “sensitivity to the suffering of oneself and others with a commitment to alleviating and preventing it” [27]. Compassion is considered to contain five elements applied to oneself and others: (1) recognizing suffering; (2) understanding the universality of suffering in the human experience; (3) empathizing with the suffering person and connecting with their distress; (4) tolerating uncomfortable feelings aroused in response to the suffering person in order to remain open and accepting of the person; and (5) motivation to act to alleviate suffering [28]. On the other hand, empathy is a form of engagement that seeks both cognitively and affectively to understand the experience of others, preserving and respecting differences [29]. This definition contrasts with compassion, which has a proactive and motivational approach to alleviating suffering [30] and does not necessarily imply a cognitive understanding of the perspectives of others [29].

Social support has been widely recognized as a mediating factor in the caregiver’s burden [31,32], acting as a protective element of mental health [17,33]. This construct is considered to be multidimensional and has been defined in various ways in the literature [34]. One of the most accepted definitions describes it as “verbal and non-verbal communication between recipients and providers that reduces uncertainty about the situation, oneself, the other, or the relationship, and attempts to improve the perception of personal control in one’s own life experience” [35]. The existing literature links social support to positive outcomes in self-rated health [36] and quality of life for informal caregivers and to a decrease in caregiver burden and negative health outcomes [37]. However, support for caregivers is inconsistent and often fails to meet their needs [38]. The aim of this study was to explore the experiences and challenges faced by informal caregivers of people with Alzheimer’s, including the social and emotional aspects of their caregiving role

## 2. Methodology

### 2.1. Study Design

A descriptive qualitative study was conducted. This approach is used to describe unfamiliar experiences, minimizing authorial interpretation and focusing on detailing both the phenomenon and the participants’ experiences in their natural state [39]. This article was written according to the Consolidated Criteria for Reporting Qualitative Research (COREQ) [40] included in Supplementary Materials.

### 2.2. Participants and Context

The study was conducted in Almería, Spain, between September 2023 and August 2024. Convenience sampling was used to recruit participants based on the following inclusion criteria: (1) being informal caregivers of older adults with Alzheimer’s disease registered as such in the Almería Health District; (2) not receiving financial compensation for care; and (3) being willing to participate in the study.

Primary care nurses collaborated in the recruitment of participants. These professionals were responsible for identifying informal caregivers who provided continuous care at home to people with Alzheimer’s disease. During routine home visits, nurses provided information about the study to caregivers who met the inclusion criteria. Those who expressed interest were referred to the principal investigator, who contacted them by telephone to explain the study objectives in greater detail and answer any questions. Thirty-six participants were invited, and 17 agreed to take part in the study. The main reasons for not participating in the study were lack of time, lack of motivation, and emotional overload on the part of the informal caregiver. No dropouts were recorded once participation had begun.

### 2.3. Data Collection

The data collection techniques used were in-depth interviews and a focus group session. First, a focus group consisting of six participants was held at a health centre in the province. Following this, eleven in-depth, semi-structured interviews were conducted at the same health centre or at the caregivers’ homes, depending on their preferences, until data saturation was achieved. This sequence allowed the researchers to delve deeper into the issues raised in the focus group and ensure no important topic was left uncovered.

The focus group lasted 120 min, and the semi-structured interviews ranged from 60 to 90 min. The interviews began with the question “Tell me about the experiences of suffering you have every day while caring for your family member.” The script for the semi-structured interviews is included in Appendix A, with the questions ordered from most general to most specific. Both the focus group and semi-structured interviews were made by two female researchers: a nurse with a doctorate and experience in qualitative research, and a novice nurse researcher with more than ten years of experience in primary care. The first was responsible for conducting the individual interviews and the focus group, and the second was responsible for recording and logging any observations and incidents. In some home interviews, the person being cared for was present, although they did not participate in the conversation; there were no other companions or people outside the study during the sessions held at the health centre.

All interviews and the focus group were audio-recorded and transcribed verbatim manually by the research team, maintaining the participants’ original expressions, pauses and emotional reactions. No linguistic cleaning or grammatical correction was performed, except for removing identifiable personal data to ensure anonymity. The manual transcription process presented several challenges, including background noise, overlapping voices, and soft-spoken participants, which required repeated listening to ensure accuracy. Fidelity checks were conducted on unclear or ambiguous sections. Before the start of data analysis, participants were invited to review their interview transcripts, clarify any points, and provide any additional information they considered relevant. Data collection was stopped when the accounts of the last three participants did not provide new insights and merely repeated what had been previously stated [41]. Therefore, analysis confirmed data saturation after 11 interviews, once no new themes emerged from the narratives. Data saturation was discussed and agreed upon by the research team before completing the data collection.

The fieldwork was affected by several methodological and practical obstacles inherent in researching caregivers with a heavy care burden. Scheduling interviews required considerable flexibility, as participants did not have enough time to conduct in-depth interviews and/or participate in the focus group. Some interviews were delayed due to the caregivers’ heavy care burden, in addition to medical appointments and their work outside the home. Other interviews were interrupted by the worsening condition of the person being cared for, forcing the researcher to reschedule them. Emotional vulnerability was also observed; on multiple occasions, participants expressed distress or cried, prompting the researcher to adopt a sensitive, trauma-informed approach and allow for breaks. These challenges required continuous adaptation of the research strategy but ultimately contributed to a richer understanding of the caregiving context.

### 2.4. Data Analysis

The ATLAS.ti software program was used to analyze the hermeneutical unity formed by the transcripts of the focus groups and individual interviews, as well as the field notes taken by the researchers. Two researchers participated in the coding and analysis process. Thematic analysis followed the steps described by Braun and Clarke [42]: (1) Data familiarization: The researchers read the transcripts to gain a general understanding of what participants had described; (2) Initial code generation: The researchers chose representative quotes and assigned codes for interesting features using the “in-vivo coding”, “open coding” and “apply codes” functions in ATLAS.ti; (3) Generation of initial themes from data coding: The researchers generated initial themes by grouping codes with similar meanings that were linked through a central idea; (4) Development and revision of themes: The two researchers reached consensus on themes and subthemes after independently checking that all themes and subthemes were consistent across codes and quotes; (5) Definition and naming of themes: The researchers reviewed the resulting themes, refined them and created final names for the themes; (6) Report writing: The researchers selected the most significant quotes and synthesized the descriptions of each theme and subtheme. Inter-coder agreement was ensured through iterative discussion and negotiated consensus, consistent with reflexive thematic analysis. The few discrepancies that arose—mainly concerning the interpretation of specific codes or the grouping of related codes—were resolved through joint review of the excerpts and refinement of code definitions until full agreement was reached.

### 2.5. Rigour

To ensure scientific rigour throughout the study, the following quality criteria were applied: (1) Credibility: the data collection process was detailed; data interpretation was supported by inter-researcher verification, and the analytical process was reviewed by two independent reviewers; (2) Transferability: the study setting, participants, context, and method were described in detail; (3) Trustworthiness: the interpretation was reviewed by an expert who was not involved in data collection or analysis; (4) Confirmability: all researchers read the transcripts independently to ensure agreement on meaning units, themes, and emerging subthemes. The research team triangulated the data analysis [43].

### 2.6. Ethical Considerations

The study was approved by the Ethics and Research Committee of the Department of Nursing, Physiotherapy, and Medicine at the University of Almería, under registration number EFM 138/2021. Participants received an information sheet explaining the nature of the study, the voluntary nature of their participation, and the guarantee of confidentiality and anonymity. Participation in the study was confirmed by signing an informed consent form.

Data processing was carried out in accordance with the Personal Data Protection Act [44] and the ethical principles of the Declaration of Helsinki [45]. The data were stored in a secure folder accessible only to the researchers, and participants were assigned pseudonyms.

## 3. Results

Seventeen informal caregivers participated in this study, 11.76% of whom were men and 88.24% women. The participants’ ages ranged from 49 to 83 years (mean 67.5, SD: 10.06). In terms of the relationship between caregiver and patient, 47.06% of caregivers were spouses, 41.18% were sons or daughters, and 11.76% were sons-in-law. Regarding the caregiver’s occupational activity, 41.17% were either housewives or retired or pre-retired, while 17.65% were actively employed. The sociodemographic characteristics are presented in Table 1.

Participants were residents of urban and peri-urban areas in the Almería Health District. Most lived in established urban neighbourhoods with access to primary care centres and social services, while a smaller group resided in peri-urban areas characterized by greater physical distance from health centres and fewer community resources. All participants lived in residential buildings. Care was provided in homes that were rarely adapted to the specific needs associated with Alzheimer’s disease. Although all buildings met basic accessibility standards (ramps and/or elevators), the interior of the homes often lacked adequate technical aids, such as hospital beds, transfer supports, or adapted bathrooms. Several caregivers reported difficulties related to limited space, the absence of safety features (e.g., grab bars), and the challenges of performing physically demanding caregiving tasks. These environmental limitations contributed to the physical burden described by participants. Caregivers’ educational levels ranged from primary to secondary education, and household income was generally low to middle, reflecting limited socio-economic resources.

Caregivers reported limited use of formal and community services. In this regard, caregivers reported that all Alzheimer’s patients received routine home visits and medical follow-up from the Andalusian Health Service. In addition, some had teleassistance and/or home help services. Only three caregivers had contact with Alzheimer’s associations and used day centres or family respite services.

Two main categories and nine subcategories were identified after data analysis, which helped us explore and describe the caregivers’ experiences and perceptions (Table 2).

## 4. Life Centred on Compassionate Care for the Other Person

This category details how caregivers adopt a way of being in the world deeply marked by emotional commitment, compassion, dedication, and empathy toward those in need of care. Amidst difficulties, many find motivation, a source of meaning and personal fulfilment, as well as satisfaction in the act of caring for their family member.

### 4.1. Compassion as an Emotional Expression of Love, Sadness, and Pity

From the perspective of informal caregivers, compassion is deeply linked to the emotional bond they maintain with the person they care for. It is not only a disposition toward care but also an emotional experience born of love, manifested in feelings of sadness and pity. For these caregivers, caring involves sharing the suffering of another, feeling sadness at the deterioration or pain of a loved one, and also a need to offer affection as a form of comfort. In this context, compassion is seen as a genuine emotional response that drives them to care from the heart.

*“I have love, to give him a lot of love, and that for me is compassion, love…”*

(GD1)

*“When he suffers, I suffer too… it’s as if his pain were mine.”*

(E10)

*“It’s not out of obligation, it’s out of love… it comes from my heart to be with her.”*

(E9)

### 4.2. The Internal Process of Accepting the Situation

The caregivers demonstrate a clear assimilation of their role, adopting it as an inseparable part of their daily lives. This acceptance is seen as an internal process that leads them to embrace the situation from a realistic perspective without any resistance. This attitude of acceptance is the first step toward building a caring relationship based on compassion.

*“This is my life, this is what we’re experiencing, this is what has happened…”*

(E4)

*“I have accepted the situation; it’s part of my life…”*

(E6)

### 4.3. The Motivations to Continue Caring

The motivation to take on the role of caregiver emerges as a network of both internal and external factors, deeply influenced by personal values, family beliefs and social circumstances. In the participants’ accounts, this motivation is not an isolated or rational decision but rather an emotional and moral construct that gives meaning to their involvement in caregiving.

One of the most recurring motives is the sense of moral obligation, where caregiving is not a choice but an inescapable responsibility, accepted with resignation and dedication. This commitment is expressed in phrases that reflect a lack of alternatives.

*“I can’t leave here because my mother is in a wheelchair, and I have to take care of her.”*

(E6)

*“Knowing that someone needs you and you have to be there because you have no other choice.”*

(E7)

These words reveal how filial duty and the lack of external support place the caregiver in a position of having an inevitable burden rather than a free choice.

On the other hand, some participants point to a motivation driven by a genuine desire to do good, to care for others with love, voluntary dedication and personal ethics. In this sense, the act of caring not only responds to the needs of others but also to an internal principle of goodness:

*“I believe I always do what I do with the best intentions.”*

(GD4)

*“I care for them because I love them; I don’t expect anything in return, only to alleviate their suffering a little.”*

(E8)

These statements reveal a search for positive meaning in the experience of caregiving, which allows them to maintain their daily tasks despite the difficulties. Furthermore, many women recognize themselves as the fundamental pillar of the family system, a role they take on with pride but also with constant pressure not to falter. The perception of being indispensable permeates their discourse and reinforces their commitment:

*“At home, I’m the one who takes on everything.”*

(FG10)

*“I tell myself, if I fall ill, this house will fall apart.”*

(FG10)

*“I need to be strong for everyone, for myself, for everyone else.”*

(E4)

*“If I’m not strong, who will take care of her?”*

(E8)

Taken together, these accounts show that the motivation to care is not homogeneous but rather responds to a combination of duty, affection, and a sense of purpose and also to social structures that place caregiving almost exclusively on women, often without a support network.

### 4.4. The Need to Develop Empathy

Empathy is presented as a key emotional tool for caregivers. In their accounts, they show a deep emotional connection with the family member’s pain, which, in many cases, also generates anguish. This empathy, although it humanizes the relationship, can generate a type of shared suffering that affects the caregiver’s emotional well-being. During the interviews, participants expressed how difficult it was to witness the physical and mental deterioration of their family members caused by Alzheimer’s.

*“I wonder how it is that a person who has been so active is now deteriorating and doesn’t talk to you about the things we have to do. I suffer and I put myself in their shoes and ask myself: How would I be?”*

(E8)

*“For me, it’s fundamental. I am very empathetic with this and with everyone. Whenever I see someone like that, I always think, ‘What would I do if it happened to me?’”*

(E6)

*“Like a mirror in which you see your reflection, in which you see yourself reflected… I try to encourage that person… I try to encourage them… but I see myself reflected in their mirror, that’s how it is.”*

(E7)

### 4.5. Committed to Alleviating Suffering

The determination to alleviate the suffering of a loved one translates into an active and committed attitude, where the caregiver gives of herself with dedication and care. Some participants describe this with complete conviction and determination:

*“I think so, it’s just that it’s not so much a question of compassion as of determination.”*

(GD6)

*“You have to treat her well, you can’t yell at her, you have to do it with love and great control.”*

(E8)

### 4.6. The Satisfaction Which Caring Gives

Finally, many caregivers find in the act of caring a source of personal and emotional satisfaction, which provides them with meaning, pride, and happiness, despite the difficulties. This positive emotional component constitutes a protective factor that cushions the impact of burnout and promotes resilience.

*“I am very happy to have him with me, and I feel strong having him.”*

(E3)

*“I also feel happy despite all the problems I have.”*

(GD2)

*“In this case, with my mother, I am enjoying being with her very, very much…”*

(GD5)

## 5. The Abandonment of the Caregivers

The analysis of these testimonies reveals that, as informal caregivers taken on the burden of long-term caregiving, they experience multiple forms of abandonment that profoundly impact their emotional, physical, and social well-being. Caregivers express feelings of progressive isolation from their family, social, and institutional environments, as well as personal abandonment, renouncing their own life plans, interests, and self-care. This accumulation of losses generates a state of pain, suffering, and emotional exhaustion that permeates all areas of their lives.

From the analysis of their statements, three major areas of abandonment emerge:

### 5.1. Abandonment by Relations and Family

Caregivers report a strong sense of loneliness and lack of support from their families and social circles. Caregiving responsibilities become a burden that is often taken on alone, without the expected support of siblings, children, or friends. These statements reflect how the caregiver is disconnected from her traditional support network, which deepens the feeling of isolation.

*“I feel alone… because sometimes I feel unwell, because of my situation… that I’m unwell, and I tell my sister, but they don’t… they each go their own way.”*

(E11)

*“I really like to travel, I really like to go out… but in that sense, I lack the support of my friends.”*

(E6)

### 5.2. Abandonment by Institutions

Another dimension of perceived abandonment relates to the lack of institutional support. Caregivers feel that public policies and social services do not adequately respond to their needs, generating feelings of helplessness and social invisibility. This institutional void leaves caregivers in a vulnerable situation, exacerbating their emotional and material burden.

*“I feel helpless because no one takes care of us.”*

(E6)

*“I don’t have any help from the social services; I feel alone in this difficult situation…”*

(E9)

### 5.3. Self-Abandonment

Caregiving ends up pushing caregivers’ own needs into the background. Thus, they cease to prioritize their well-being, hobbies, and personal development, negatively affecting their identity and quality of life. This progressive abandonment reinforces the emotional burden caregivers face, leading them to a state of physical, emotional, and social exhaustion that urgently requires recognition and support. Furthermore, abandonment is not a one-time experience but rather a progressive and silent process that affects multiple dimensions of caregivers’ lives.

Social isolation appears as one of the first signs as they are forced to give up group activities, personal relationships, and community development spaces.

*“I was a very active woman; I used to take photography and painting courses… now all that is very limited, it’s nothing at all.”*

(E6)

*“I miss many things, I don’t have a social life…”*

(E6)

Added to this is the deterioration of self-care: many caregivers admit to having neglected their physical appearance, their diet, or their health routines.

*“Now I’ve really neglected my physical appearance; I really don’t look after myself.”*

(E5)

*“I’ve been losing a lot of weight; that’s what comes with devoting myself 100% to caring for others…”*

(E10)

They also show a reluctance to pursue their interests, hobbies, and interests, those minor activities that once brought them enjoyment and a sense of well-being. All of this is transforming their lifestyle, now limited by confinement, routine, and the emotional burden of caregiving.

*“I don’t have time or peace of mind to read.”*

(E10)

*“I used to dance, go to the gym… but now I need my space, which I don’t have.”*

(E7)

*“Because we went out with friends, we used to go to the theatre, to concerts… all of that has been lost. We don’t go out to eat anymore, we don’t go out with friends, we just stay at home…”*

(E10)

Finally, this accumulation of renunciations leads to the abandonment of their own life project: they stop living for themselves and live exclusively to support others. Below are personal testimonies that reveal this process.

*“I don’t do anything for myself, I do what I do every day, I certainly don’t do anything for myself. I exist, and that’s it.”*

(E9)

*“Before, I had dreams, plans for the future… and now I only live to take care of her.”*

(GD3)

## 6. Discussion

The aim of the study was to explore the perceptions and opinions of Alzheimer’s caregivers regarding compassion and social support. The sociodemographic characteristics of the participants in our study were similar to those previously described [46,47], with the majority being women, daughters, or partners of the patient, and with low academic levels. More specifically, the local context (Almería) must be interpreted within the Mediterranean model of care and the specific socioeconomic and political dynamics of the region. As in other southern European countries, long-term care provision in Spain has traditionally been based on informal family support, provided predominantly by women and supplemented only by occasional formal services [48]. These structural weaknesses mean that the public long-term care system serves only a limited part of the population, leaving most of the responsibilities in the hands of informal caregivers and migrant workers, often employed in irregular conditions [49]. This pattern is consistent with the Mediterranean context, where informal care is widespread and public benefits are comparatively low, unlike the systems in northern and central Europe, which offer more generous benefits and rely less on family care [50]. Regional factors further intensify these structural pressures in Almería. The implementation of Spain’s Dependency Law has been affected by delays and resource constraints [51]. As a result, caregivers bear significant health, social, and work burdens, despite growing evidence of the need for respite policies and more robust formal care provision [52]. The local context of Almería therefore reflects the sociodemographic patterns of southern Europe and the specific limitations of the region, reinforcing the Mediterranean model of family-based care.

Compassionate care emerges in caregivers’ discourses as a key element in patient care. Previous studies have shown that this type of care can be more beneficial to patients than other aspects such as empathy [30], in addition to helping to reduce the caregiver burden [30,53]. The experience of caregiving can be positive or negative [17]. If the experience is perceived as positive, it can increase self-esteem and improve personal relationships, which is a crucial aspect for informal caregivers [54]. However, it can be a negative experience when the caregiver’s resources and needs are unbalanced, decreasing life satisfaction [55].

On the other hand, we found that caregivers in our study had misperceptions about the concept of compassion. This may be because the concept of compassion is complex and difficult to define [28] and bears similarities to other constructs such as empathy or sympathy [56]. Furthermore, participants reported the importance of accepting their life situation. It has been observed that caregivers of Alzheimer’s patients experience a progressive process that begins with denial (fear, anger and/or anxiety) and ends with acceptance [57]. Furthermore, lack of acceptance by caregivers can represent a significant obstacle in the care of patients with Alzheimer’s [58].

In our study, we identified a strong moral and cultural burden among caregivers’ main motivations. These findings are consistent with the meta-ethnographic review by Zarzycki et al. [59], which highlights cultural and familial components, as well as feelings of obligation and reciprocity, as common factors in caregivers’ motivations. Furthermore, our participants emphasized a desire to do good, offering care voluntarily and finding personal pride in it. In this sense, Li et al. [60] found that the will to care is closely linked to altruism and is related to factors such as responsibility, love, and the quality of the relationship. However, altruistic care can be idealized and romanticized, potentially generating a high demand for self-sacrifice [61]. Regarding the pride felt by our participants, this emotion is often related to the pursuit of achievement, boosting motivation, strengthening social authority, and fostering personal growth [62]. This characteristic has been considered a positive aspect of caregiving, linked to positive thinking, reducing negative emotions, and promoting caregiver resilience [63]. Caregiver abandonment also emerged as a constant among our participants. Caring for a person with Alzheimer’s requires high levels of empathy, which can generate negative feelings when the suffering of others is indistinguishable from one’s own [64]. Although empathy has been linked to positive aspects such as stress reduction, if self-regulation strategies are lacking, it can lead to emotional distress [24]. Our study also clearly reflects the perception of abandonment by institutions and the family environment. This situation has been widely documented, and it has been proven that the lack of social support contributes significantly to the increase in emotional and physical overload of informal caregivers [17,65]. The absence of social support contributes to the progressive abandonment of themselves, as well as their social relationships, their hobbies, their self-care and their life project [66], producing a decrease in the quality of life and increasing physical and mental health problems [67]. Scientific evidence highlights that compassion-based interventions [68] and the strengthening of support networks could significantly mitigate the emotional burden of informal caregivers [69,70].

This study contributes novel insights by showing how compassion, moral obligation, altruism, pride, and institutional abandonment interact simultaneously in Alzheimer’s caregiving, a combination not previously described. It also identifies a conceptual misunderstanding of compassion among caregivers and reveals how positive motivations can coexist with progressive self-abandonment, offering a more integrated perspective on the caregiving experience. However, this qualitative study has certain limitations. While the sample reflects common sociodemographic profiles of informal caregivers, it is not statistically representative, and the findings cannot be generalized to the entire population. The study prioritizes depth and richness of experiences, focusing on transferability to similar contexts rather than broad statistical generalization. A higher proportion of male caregivers, inclusion of cultural factors, and participation from rural areas could have added further perspectives. Moreover, the participants were interviewed only once. Additionally, conducting more focus groups could have facilitated peer interaction and the exploration of multiple viewpoints, potentially enriching the data. Finally, a limitation of this study is that, although participants reviewed their interview transcripts, no formal member checking of the final findings was conducted. Future studies should consider including these aspects.

## 7. Conclusions

The results show that informal caregivers take on a compassionate role, deeply marked by love, commitment, empathy and the satisfaction that comes from helping their family member. However, this commitment entails a high personal cost, reflected in the abandonment of their own selves, their support networks, their physical and emotional well-being, and their life plans. This duality highlights the need to understand caregiving not only as an individual and emotional act but also as a shared social responsibility. An urgent review of public policies and the current care model, which continues to place the burden of care on informal caregivers without providing adequate support, is therefore needed. It is essential to promote psychosocial interventions that include compassion, as well as the strengthening of formal and informal support networks. Only through these measures will it be possible to transform caregiving into a sustainable, dignified, and healthy experience, thereby enhancing the quality of life and emotional well-being of both the care recipients and their caregivers.

## Figures and Tables

**Table 1:** Sociodemographic characteristics of the participants.

Participants	Sex	Age	Marital Status	Relationship	Cohabitant	Caregiving Shared with Other Family Members	Work Outside the Home
P1 (E1)	F	58	Married	Daughter	Yes	No	Nursing Care Technician
P2 (E2)	F	55	Separated	Daughter	Yes	No	Home Care Service
P3 (E3)	F	76	Married	Spouse	Yes	No	Housewife
P4 (E4)	F	83	Married	Spouse	Yes	No	Housewife
P5 (E5)	F	70	Married	Spouse	Yes	No	Retired
P6 (E6)	F	69	Widow	Daughter	Yes	No	Retired
P7 (E7)	F	62	Married	Daughter	No	Siblings	Housewife
P8 (E8)	M	79	Married	Spouse	Yes	No	Retired
P9 (E9)	F	76	Married	Spouse	Yes	No	Housewife
P10 (E10)	F	80	Married	Spouse	Yes	No	Retired
P11 (E11)	M	63	Married	Son-in-law	No	Spouse	Early Retired
P12 (GD1)	F	66	Married	Daughter	No	Siblings	Retired
P13 (GD2)	F	49	Single	Daughter	Yes	No	Administrative
P14 (GD3)	F	79	Married	Spouse	Sí	No	Housewife
P15 (GD4)	F	64	Married	Daughter	Sí	No	Housewife
P16 (GD5)	F	63	Married	Spouse	Yes	Daughter	Housewife
P17 (GD6)	M	56	Married	Son-in-law	Yes	Spouse	Early Retired

M: male, F: female.

**Table 2:** Units of meaning, subcategories, and categories.

Units of Meaning	Subcategories	Units of Meaning
Compassion, love, sadness, shared pain, tenderness, “from the heart”, consolation, suffering, emotional bond	Compassion as an emotional expression of love, sadness and pity	Life centred on compassionate care for the other person
Resignation, “this is my life”, lack of resistance, routine, internal process, accepted role	The internal process of accepting the situation	
Moral obligation, filial duty, “there is no other way”, personal values, affection, being the family pillar	The motives for continuing caregiving	
Reflection, mirror effect, “putting yourself in their shoes”, emotional exhaustion, emotional connection	The need to develop empathy	
Determination, patience, moderation, affection, support, emotional presence	Committed to the relief of suffering	
Accomplishment, pride, inner strength, “joy of caring,” resilience, emotional reward	The satisfaction that caring gives	
Loneliness, “no one helps me,” distancing from friends, single responsibility, lack of support	Abandonment by relations and family	Abandonment of the carers
Lack of resources, “invisible”, lack of social services, bureaucracy, lack of protection	Abandonment by the institutions	
“No time for myself”, physical neglect, loss of hobbies, “I just exist”, tiredness, sadness	Abandonment of themselves	

## Data Availability

The datasets generated and analyzed during the current study are not publicly available due to privacy and confidentiality agreements with participants, but are available from the corresponding author on reasonable request (in Spanish language).

## Supplementary Materials

The following supporting information can be downloaded at: https://www.mdpi.com/article/10.3390/healthcare13243271/s1, COREQ (COnsolidated criteria for REporting Qualitative research) Checklist.

## Appendix A. Interview Guide of Semi-Structured Interview

Pregunta de arranque: Cuéntame las vivencias de sufrimiento que tienes día a día en el cuidado de tu familiar.

Q1: ¿Cómo es el sufrimiento de la persona que cuidas? ¿Y el tuyo?
Q2: ¿Cómo te sientes ante el sufrimiento del familiar al que estas cuidando?
Q3: ¿Cómo responde tu cuerpo, tu mente, tus emociones?
Q4: ¿Cómo actúas ante el sufrimiento de tu familiar? ¿Cómo te sientes después?
Q5: ¿Qué sientes que necesitas para poder relacionarte con el sufrimiento de manera sana? Y ¿que necesitas para sostener tu propio bienestar?
Q6: Desde tu punto de vista, ¿qué importancia le das a ponerte en el lugar de la persona a la que cuidas?
Q7: ¿Qué te ayuda a ponerte en el lugar de tu familiar? Y, ¿Qué te dificulta?
Q8: ¿Tener compasión es importante para tú? ¿Por qué?
Q9: ¿Has sentido alguna vez que no puedes ser compasiva con tu familia? ¿Cómo fue esa experiencia?
Q10: ¿Cómo te tratas a ti misma?
Q11: ¿Crees que eres compasiva contigo misma? ¿Por qué?
Q12: ¿Podrías contarme como te sentiste en alguna ocasión en la que hayas intentado hacer algo y no te salió bien?
Q13: Cuéntame… ¿te juzgas a ti misma?, ¿de qué manera?
Q14: ¿Cuánto de sola o acompañada te sientes en esta situación?
Q15: ¿Cuándo ves a otras personas viviendo situaciones iguales o similares a la tuya, como sientes?
Q16: Cuéntame cómo afrontas la situación que estás viviendo.
Q17: ¿Piensas que tienes recursos para adaptarte a esta situación?
Q18: ¿En qué o en quien te apoyas?
Q19: ¿Qué te ayudaría a afrontar esto que vives de una manera más sana para ti?
